# AI-powered narrative building for facilitating public participation and engagement

**DOI:** 10.1007/s44163-022-00023-7

**Published:** 2022-03-30

**Authors:** Fernando Marmolejo-Ramos, Thomas Workman, Clint Walker, Don Lenihan, Sarah Moulds, Juan C. Correa, Anca M. Hanea, Belona Sonna

**Affiliations:** 1 Centre for Change and Complexity in Learning, The University of South Australia, Adelaide, SA 5000 Australia; 2 Converlens Pty Ltd AU, Melbourne, Australia; 3 Middle Ground Policy Research CA, Ottawa, Canada; 4 UniSA Justice & Society, The University of South Australia, Adelaide, Australia; 5 CESA Business School, Bogotá, Colombia; 6 Ecosystem and Forest Sciences, University of Melbourne, Melbourne, Australia; 7 African Institute for Mathematical Sciences, Kigali, Rwanda

**Keywords:** Narrative building, Artificial intelligence, Governance, Public affairs, Natural language processing, Expert knowledge elicitation

## Abstract

Algorithms, data, and AI (ADA) technologies permeate most societies worldwide because of their proven benefits in different areas of life. Governments are the entities in charge of harnessing the benefits of ADA technologies above and beyond providing government services digitally. ADA technologies have the potential to transform the way governments develop and deliver services to citizens, and the way citizens engage with their governments. Conventional public engagement strategies employed by governments have limited both the quality and diversity of deliberation between the citizen and their governments, and the potential for ADA technologies to be employed to improve the experience for both governments and the citizens they serve. In this article we argue that ADA technologies can improve the quality, scope, and reach of public engagement by governments, particularly when coupled with other strategies to ensure legitimacy and accessibility among a broad range of communities and other stakeholders. In particular, we explore the role “narrative building” (NB) can play in facilitating public engagement through the use of ADA technologies. We describe a theoretical implementation of NB enhanced by adding natural language processing, expert knowledge elicitation, and semantic differential rating scales capabilities to increase gains in scale and reach. The theoretical implementation focuses on the public’s opinion on ADA-related technologies, and it derives implications for ethical governance.

## Introduction

In today’s world, the existence of several initiatives aiming to harness the benefits of algorithms, data, and Artificial Intelligence (ADA)^1^ reveals a global interest shared by both public and private organizations. In the private sector, “Partnership on AI” emerged as a non-profit coalition committed to the responsible use of AI and have included large corporations such as Amazon, Deepmind, Microsoft, IBM, Google, Facebook, or Samsung, among many other actors like academic associations, research institutions, and even start-ups. In the public sector, “Open Government Partnership” emerged as an organization of reformers inside and outside of government working to transform how government serves its citizens, and have gathered around seventy-eight countries and seventy-six local governments, representing more than two billion people along with thousands of civil society organizations. As recent reviews have covered this topic for the private sector (e.g., [12, 44]), this manuscript focuses on ADA for the public sector in general, and the digital governance (DG) in particular.

According to Erkut [28], DG refers to a process encompassing the design and use of digital government, digital business issues, and digital democracy as a process that transcends the mere concept of providing government services digitally. In other words, DG aims to change the nature of establishing and running a business as well as the democratic representation of people. Open Government Partnership, for example, included DG among one of its policy areas.^2^ The DG’s goal is to have governments using any form of ADA technology to be more transparent, open, accountable, and inclusive.

Nowadays, several national governments have embraced these ideas in “action plans” (e.g., Australia,^3^ France,^4^ Canada,^5^ UK,^6^ USA,^7^ Germany,^8^ Colombia,^9^ among many other countries). Given the societal role of ADA-related technologies [6], these governmental plans seem to be reasonably sensitive to the changes associated with the so-called Industry 4.0, i.e., the automation of processes via smart technology [84]. Along with these action plans, however, other challenges have been foreseen. For example, the French government has claimed the need for a better understanding of the issues and potential risks involved in using algorithms in the management of public action.

Machine learning algorithms play an important role in the implementation of public policies in the areas of public finances and education, for example. The transparency of algorithms is of vital importance in providing citizens with information on administrative decisions. In this context, it is desirable that any person who is subject to an individual administrative decision based on algorithms (i.e., human decision making augmented by AI) must be informed of the fact and may demand access to the algorithms’ main operational rules. Furthermore, source codes should be present in the list of communicable administrative documents (see also European Data Protection Board; previously known as Article 29 Working Party). This speaks to one of the foundational principles in administrative law: procedural fairness. Any citizen who is impacted by an administrative decision—for example where that decision relates to the amount of social security they receive or their eligibility for a visa—is entitled to understand *how* that decision has been made so they can check that it was made *lawfully* [67], p. 47–50). Enabling access to source codes is an important component of understanding the decision-making process when machine learning algorithms are used in administrative decision making [49].^10^

In this paper, we introduce the term ‘narrative building’ (NB) as a tool aiming at facilitating public engagement with these digital governance initiatives through ADA-related technologies. Our central idea posits that NB aims to overcome two major problems of DG: scale and reach. By ‘scale’ we mean increasing the number of individuals that engage with government decision making. By ‘reach’, we mean reaching new audiences beyond the ‘usual suspects’ (for example, experts, inner city well educated, industry groups) to include those with more diverse lived experiences, including those located in regional and remote locations, or from non-English speaking backgrounds. This article aims to explore these relationships by (1) describing the conventional forms of public engagement that are employed by governments seeking to engage with the citizens they represent and (2) describing how narrative building techniques can be combined with ADA related technologies to improve the scale, reach, diversity, and deliberative quality of those engagements. In doing so, NB is exemplified as a natural form of public engagement and deliberation and then some ADA-related technologies in society and government are considered. Finally, the basic pillars of what an AI-powered NB could be like are sketched in relation to the issue of public’s perceptions of ADA technologies and related implications for governmental decision-making and governance are considered. We would like to emphasise that our proposal is a theoretical one that will need to be empirically validated to gauge its true potential.

## Limitations of traditional forms of public engagement employed by governments

In the modern parliament, public engagement is essential for effective parliamentary scrutiny and to ensure that parliament remains relevant to society [72]. So too when it comes to the workings of government and public administration, which is “*increasingly concerned with placing the citizen at the centre of policymakers’ considerations, not just as target, but also as agent*” [35], p. 1). The growing realisation of both the benefits (e.g., [60]) and necessity (e.g., [20]) to engage the public in the process of lawmaking and policy making has led to parliaments and governments around the world investing a range of different public engagement activities and modalities (e.g., Organisation for Economic Co-operation and Development, 2009).

**Fig. 1:**
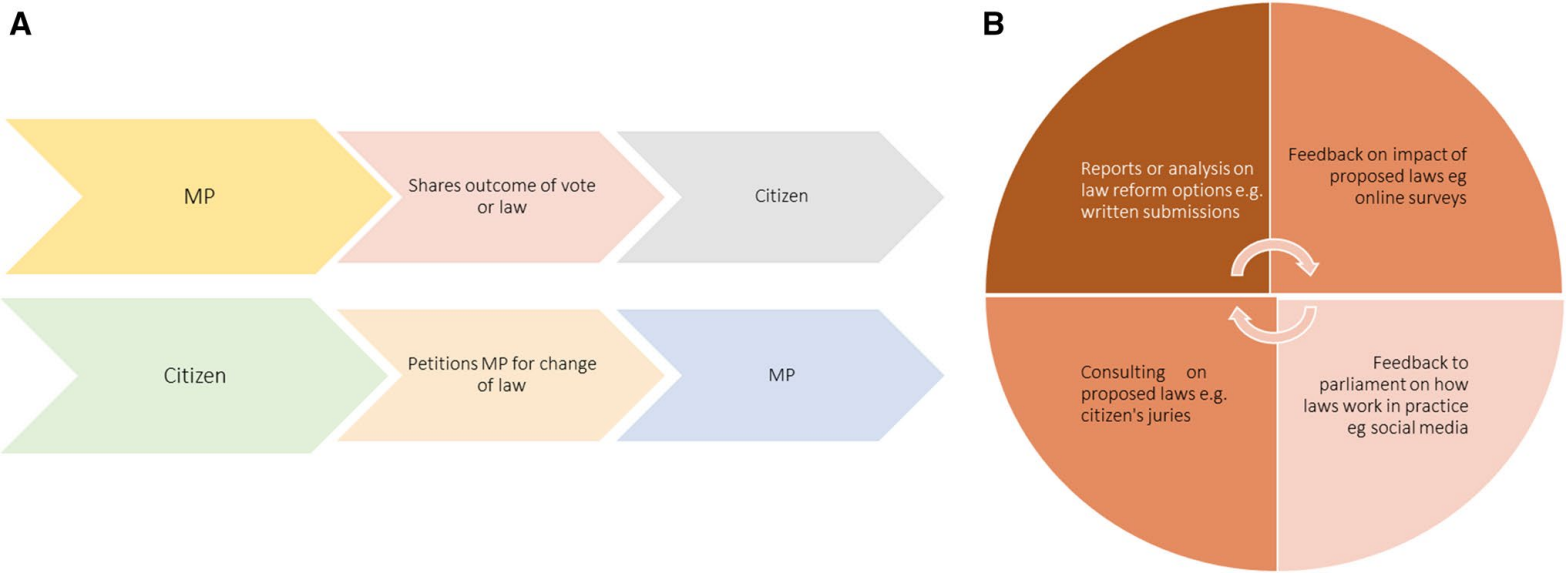
Types of public engagement. Public engagement as two types of one-way information sharing (**A**) and as a deliberative process (**B**)

Public engagement techniques have traditionally focused on one-way information sharing [89], but in recent decades, there has been a decided shift towards more deliberative participation in the policymaking [35] and law-making processes [47] (see Fig. 1). This shift towards more deliberative forms of engagement has led to important modifications being made to ‘conventional’ public engagement strategies (such as inviting a more diverse range of stakeholders or communities to participate in public hearings or to submit written submissions) and encouraged innovative new engagement techniques (such as the establishment of online forums or platforms such as the YourSAy site established by the South Australian Government).

Some of the impacts of public engagement (for example, changing the content of the law or increasing the diversity of participation in a public forum) may be easier to quantify than others (such as changing the culture within a government department). Some forms of public engagement (such as tracking the passage of a proposed law) may be easier to ‘digitalise’ than others (such as consulting on a complex policy).

Digital engagement techniques, such as secure video conferencing and legislation tracking apps, have become increasingly attractive to parliaments with the potential to improve both the quality and efficiency of public engagement in lawmaking, leading to cost savings and possible improvements in democratic participation [42, 61]. Scholars in other jurisdictions (e.g. [23, 36, 60]) have begun to document the democratic and economic benefits of using certain digital tools (such as e-petitions or legislative tracking apps) to facilitate public engagement, and some have also identified risks associated with their use (e.g. [81]).

Other scholars, such as Macnaghten and Guivant [50] and Prior and Leston-Bandeira [72] have explored the important role story-telling and narratives play in both designing and facilitating effective public engagement strategies, and when it comes to improving citizen’s understanding of emerging public policy challenges and potential solutions. For example, Macnaghten and Guivant [50] have explained that using ‘situated narratives of epistemic inclusion’ is essential to developing innovation frameworks that will be appropriated and adapted to the societies they seek to serve. Similarly, Prior and Leston-Bandeira [72] have observed that the use of storytelling techniques, that include narrator, characters plot and audience, provides a critical opportunity to represent Parliament and government as a relevant and relatable institution, and a safe space for meaningful public engagement.

Ultimately, as Holmes foreshadowed in 2011 [35], effective public engagement in policy and law-making processes requires the “genuine devolution of power and decision-making to frontline public servants and professionals—and to the citizens and stakeholders with whom they engage”. Recent scholarship cited above suggests [63, 72] that public engagement modalities that include a strong focus on inclusion, deliberative engagement and narrative discourse provide a practical pathway for such devolution of power, without disrupting the important democratic and political lines of accountability that must be maintained between the people and their elected representatives.

As explored further below, when coupled with narrative building techniques, ADA-related technologies offer important new opportunities to adapt conventional engagement strategies to reach new and larger audiences, and to encourage new innovations in public engagement experiences. This primarily because the combination of these two different modalities delivers both legitimacy and accessibility for the citizen. Legitimacy comes from the use of storytelling techniques that position the citizen in the centre of the conversation, allow them to 'be heard' and empower them to contribute to policy making in a deliberative and meaningful way. Accessibility comes from the use of ADA-related technologies that can bridge the cultural, geographic and resource divide that has operated to exclude certain communities from being able to access government decision making in the past.

The need to embrace legitimate and accessible public engagement strategies cannot be overstated, particularly in the wake of the COVID-19 pandemic which has fundamentally reshaped the relationship between citizen and government in democracies around the world. If citizens do not understand what their governments propose with new legislation rules, then democratic participation might not be entirely fair or justified. An example of this phenomenon occurred in 2016 in Colombia [18]. Back in that time, the Colombian government proposed a plebiscite to estimate the public support of a peace agreement with the guerrilla group FARC to finish the 52-years-long Colombian armed conflict [18]. The plebiscite counted 62.57% of voters’ abstention, and less than 20% of the Colombian electorate rejected this agreement [18]. According to Correa et al. [18], this outcome was concomitant with the text difficulty of this peace agreement as it was not written for broader and less-educated audiences.

In sum, for DG initiatives to be successful, policymakers need to facilitate the public comprehension of governmental decisions. As it becomes clearer later, narratives are a driving mechanism to achieve such a goal by being enhanced with other data processing techniques such as expert knowledge elicitation, natural language processing, and semantic differential rating scales.

## Narratives and NB: psychological basis and social role in public engagement

### NB’s psychological basis

Research in discourse processing has investigated how people comprehend (and produce) both written and oral texts or discourse [56]. There are three types of discourses: expository, argumentative, and narrative [56]. Expository texts aim at explaining a topic, scientific and academic articles are exemplars of this type of texts. Argumentative texts present arguments in relation to a topic; academic essays are typical exemplars of this types of texts, but academic writings can be both expository and argumentative. Both types of texts follow a non-narrative structure in their style. Narrative texts, contrary to other types of texts, are characterised by including characters, emotions, timelines, and space. Processing any of these three types of texts requires cognitive processes such as semantic and syntactic awareness, inferencing, and planning/organizing. In relation to narrative texts, neuroscientific work has shown that frontal lobe regions are essential in understanding this type of texts and those brain regions are associated with executive functions such as inhibition, monitoring, and planning [56] for a review).

Research, however, has shown that narrative texts are easier to process than any other type of text as this type of text/discourse is introduced early in life [54]. Also, research has shown that narrative texts enable rich mental representations that encapsulate the gist of a narrative’s topic [52]. In a nutshell, scientific evidence has indicated that narratives are the most optimal discursive tool to retain the gist of any message in memory, elicit emotional responses, and promote social cognition [45, 52, 54, 55, 56].^11^ Narratives thus aid in the goal of collectively constructing representations of reality and charge them with meaning [10]. An essential component in the construction of narratives is metaphors as these further shape cognitive processes needed for the production and comprehension of narratives. Metaphors help better reasoning and decision making by incorporating affective and social components necessarily linked to any narrative [87].

### NB’s social role in public engagement

Policy disputes are usually driven by underlying “narratives” that shape people’s views of an issue [46, 86]. Imagine climate change as a meteor hurtling toward the earth. The image can be used to guide the creation of a script or story–a narrative–that tells people how to view and respond to the crisis, from reorganizing their priorities to preparing for the damage ahead. Basically, the metaphor frames a story that gives order and meaning to a complex set of facts, values, and priorities. Table 1 illustrates how building a narrative around a guiding metaphor or image can be analysed into five basic tasks.

**Table 1:** Five key steps in narrative building using the climate change crisis in Canada as an illustration

Task	Explanation	The catastrophe view (narrative 1)	The social impact view (narrative 2)
Define the viewpoint	A narrative uses descriptive language to define a viewpoint on a situation	“Climate change is like a meteor hurtling toward the earth and the result will be a catastrophe unlike anything in history.”	“Oil and gas are the lifeblood of Alberta’s (an oil-producing province in Canada) economy; shutting the industry down would kill our towns and end our way of life.”
Anchor it in reality	A narrative also uses factual claims—which may or may not be true—to explain and support the viewpoint on the situation	“At current rates, global greenhouse gas emissions will cause a temperature increase of 4 degrees Celsius by 2050. The impact on the biosphere would be catastrophic”	“Canada’s oil and natural gas industry produces only about 0.3% of overall global GHG emissions. Shutting the industry down would have no discernible impact on climate change.”
Shape people’s response	A narrative incorporates certain societal values, interests, or priorities and uses them to define how people should think or behave in the situation	“We owe it to future generations—and every other species on the planet—to bring GHG emissions under control before it is too late.”	“The oil and gas industry is a leader in clean technology. Canada can and should capitalize on the opportunity to become a global leader in responsibly produced oil and gas.”
Exclude competing options	The viewpoint of a narrative often casts a situation in binary terms (either/or, for/against, right/wrong)	“The only way to bring global warming under control is an immediate end to the use of carbon-based energy.”	“Global demand for natural gas will increase by 29% by 2040, supplying 25% of total energy consumed worldwide, and global demand for oil will increase by 7%, supplying 28% of total energy consumed. If Canada doesn’t provide this energy, someone else will.”
Contextualise the storyline	A narrative can usually be reduced to a simplified storyline whose core elements are easily shared and can be adapted to accommodate the details of different circumstances	“Global warming is the result of callous disregard for the biosphere and rampant consumption of carbon-based energy. Renewable energies are available, and the world must transition to them immediately. Decisive government action is essential, including… (insert details)”	“Canada exported $122 billion worth of gas and oil in 2019. Rather than shutting down one of our most profitable industries down, Canadians should be working to transform it, based on a new commitment to responsibly produced energy through cleantech and a staged transition to renewal energy by… (insert details)”

Narrative 1 and narrative 2 are competing narratives [46]

Using different metaphors or images to describe a situation can generate competing narratives. Consider the debate over how aggressively people and governments should respond to climate change. The meteor metaphor suggests that the shift to green technologies should be rapid and dramatic. But if the defining metaphor focuses on the disruption that change will cause in people’s lives, it may suggest a staged or more gradual transition (see Table 1).

Policy debates like this are driven by competing narratives that organize facts and values differently and, as a result, lead to different conclusions about how people should respond to an issue. Insofar as these disagreements are over facts, they should be resolved by an appeal to evidence. Disputes over values and interests, however, must be resolved differently. They require “trade-offs” or a “balancing” of the conflicting views. The challenge is that balancing values or making trade-offs is often highly subjective and there are very few tools to help people resolve such tensions.

Typically, people are instructed to sit down together, stand apart from their subjective views and interests and try to find a reasonable and fair accommodation of their values or interests. This is not only difficult; it can be divisive. What standards should they use to guide their deliberation? Who gets to decide what counts as “fair and reasonable?”. NB makes this process more reliable and systematic. The participants work together to turn their competing narratives into an integrated, third story—a shared narrative—which reframes the issues in a way that establishes common ground and gets a productive discussion going on difficult tasks, such as balancing competing values. In the case of climate change, the participants might work together to tell a more nuanced story about the transition to renewable energy sources, one that people from both sides would find acceptable. Note though that that there will be stakeholders who are very much invested on both sides of the discussion, and this is a situation that adds complexity to deliberations around this topic.

**Fig. 2:**
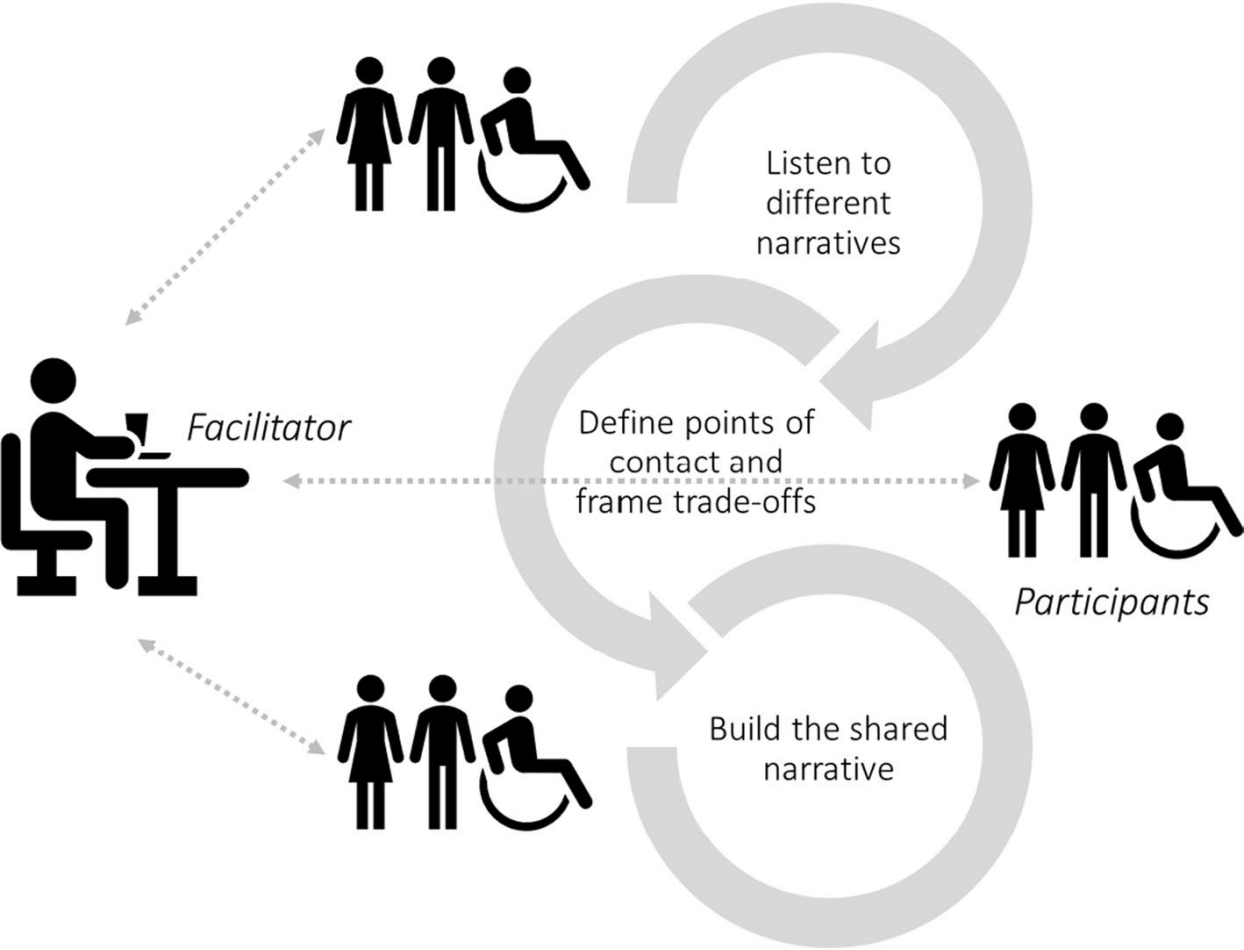
Illustration of the narrative building process

The methodology for building a shared narrative involves different kinds of give-and-take at three stages of the dialogue, each of which is defined by a different task: (i) get the participants to listen to each other’s stories, (ii) help them to explore the experiences behind the different narratives, identify points of contact between them, and define the trade-offs that must be made to resolve the issue; and (iii) guide the participants as they work together to build a shared narrative that aligns key elements of their stories in new ways. Figure 2 illustrates how the methodology works.

In sum, NB uses deliberation to create a shared story about the context around an issue. This story helps the participants recognize and understand the role that values play in their dispute and highlights other objectives or values that they share. A shared narrative thus establishes common ground and, ideally, that allows the parties to start an informed and respectful discussion of how they can accommodate one another’s views.

Based on previous illustrations, we claim that NB should be regarded as a natural form of public engagement and deliberation with the potential of being enhanced through the use of ADA-related technologies. The role of artificial intelligence in this matter is a key factor that governments can leverage in the context of the fourth industrial revolution or Industry 4.0; I4 [84].

## AI in society and government

“Incorrect. I am not an AI. My code name is Project 2501. I am a living, thinking entity that was created in the sea of information.” – Puppet Master (Ghost in the Shell)

The fourth industrial revolution is characterised by the ubiquity of information and digital technologies. This revolution is epitomised in what is known as artificial intelligence (AI); canonically understood as any type of technology that automates processes and exhibits human-like intelligence (e.g., [71]) (note, however, that current definitions of AI aim to not anthropomorphise it [75] and instead seek to differentiate it from human intelligence [39]). AI relies on data and algorithms [88] and, together, permeate many sectors of society (e.g., [76]). While AI can be defined as above, data can be defined as information about objects, events, processes, and persons that is encoded digitally [88]. Algorithms can be defined as procedures designed to perform automated tasks by using data sets and assist reasoning and decision-making. In other words, data are used to feed algorithms and algorithms are used to drive AI agents, i.e., algorithms are the ‘ghost’ in the AI ‘shell’.

AI is used to solve problems and achieve goals with limited or no human supervision. General AI aims to match human behaviour and it is forecast to occur by 2030 or thereabouts; narrow AI, instead, refers to algorithms designed to carry out specific tasks in an automated fashion [21]. This latter type of AI is the most discussed in recent years as it is one of the distinctive features of the I4. For example, algorithms are being adopted by institutions, organisations, and governments to deal with the vast amounts of data amassed by these sectors to expedite and optimise decision-making processes [27].

More generally, ADA-related technologies have been at the service of scientific progress and have been used for developing powerful analytical tools [85]. Those technologies have also been at the centre of discussions around ethical concerns regarding disinformation, discrimination, and privacy [3, 51]. In fact, factors such as accuracy, fairness, explainability, stability and adoption can affect the benefits of ADA-based solutions in the public sector [25, 85]. People’s lack of understanding of how ADA technologies work could affect adoption of these technologies and ultimately determine the degree of trust placed on them. More crucially, fine-tuning the degree of trust in ADA technologies will determine the quality of decision-making processes relying on those technologies. Thus, it becomes a priority to encourage a combination of ADA literacy with transparent digital governance for all governments and their initiatives.

Powerful algorithms designed to assist decision-making processes are already in use in several applications including healthcare and law enforcement sectors. In the healthcare sector, for example, doctors assisted by AI tools have made advances in automatically diagnosing diseases, making diagnostics cheaper and more accessible [1]. In addition, some studies made by Standigm Inc., one of the first companies applying AI tools to drug discovery and development, show that AI promises to cut the cost and timeline of drug development by eliminating some of the guesswork from the process [62]. The key of this company is blending chemistry and biology expertise with AI tools. The examples listed above highlight the potential of ADA technologies in improving the healthcare system with massive capabilities of computing technology that are efficient and low time consuming.

ADA technologies are also being used in the governance sector. For example, the Harm Assessment Risk Tool (HART) went live in 2017 in the UK with the goal of reducing reoffending rates in England and easing pressure on the criminal justice system [68]. The system was built with 104,000 cases of people arrested in custody. Even though the system is under review due to some ethical concerns, this tool has helped the government to do more with less resources in the process of custody officers [14, 64, 68], for the COMPAS, Correctional Offender Management Profiling for Alternative Sanctions, is a U.S.A. counterpart). ADA technologies have been instrumental in sifting through large amounts of documents. For example, Airbus underwent investigation to determine bribes it was paying via a middleman. There were about 500 million documents that needed to be parsed and AI was used as a technology-assisted review (TAR) [31] to cope with the task of exposing fraud [24]. TAR has also been used to facilitate civil litigation as exemplified in the McConnell Dowell v Santam (in 2016 in Australia), Pyrrho Investments Ltd v MWB Property Ltd (in 2016 in the UK), Irish Bank Resolution Corporation Ltd & Ors v Quinn & Ors (in 2015 in Ireland), and Rio Tinto Plc v Vale S.A. (in 2015 in the U.S.A.). TAR is a method within the larger field of natural language processing (NLP). In broad terms, NLP is an ADA technique that focuses on parsing vast amounts of texts to produce explainable and insightful summaries [15], and has also been shown to be highly instrumental in opinion mining, also known as sentiment analysis or emotion AI [82]. In the case of policymaking (i.e., decision making around policies), ADA is essential to identify key topics, formulate likely scenarios, generate data for substantiating decisions, expedite output delivery, and suggest adjustments and improvements to policies (see [70]). Despite the potential benefits of ADA to policymaking, there are challenges that need to be addressed. Zuiderwijk et al. [91] identified seven aspects that need consideration in relation to AI specifically, some of them are: investigating effective implementation plans and metrics for government strategies on AI use in the public sector, examining how governments can better engage with and communicate their AI strategic implementation plans to stakeholders, and researching how the performance and impact of public sectors’ AI solutions can be measured. In a nutshell, these authors recommend that multidisciplinary and theory-driven research on AI use in public governance is needed.

## AI meets NB: towards AI-powered NB

### Motivation

Narratives have permeated the political world [9] and NB as a public engagement tool is an example of this. The new twist, however, would be that ADA technologies and policymaking occur within an NB framework to enable public engagement with scale and reach^12^ (such blend between ADA technologies and policymaking is gaining momentum [38]). Given that in the case of governance complex ideas refer to policymaking, NB is a suitable too as its goal is to facilitate people to manipulate complex ideas through narratives.

NB can be categorised as a component of deliberative discussions that form part of informed participation with communities [86]. NB from this perspective has shown to have many benefits, but there are also cost barriers with the associated processes required to engage the community in this form of deliberation, primarily scale and reach. This is largely because NB requires face-to-face interactions. Face-to-face interactions require people to meet in a central location which can result in costs such as room hire, catering, travel, etc. In addition to this, timing of events can be restricted to limited periods due to travel requirements and opening hours of venues, which can further restrict the amount of people that can attend these interactions (the current COVID-19 pandemic is an example of this situation; see [73]). There are also many areas of the community that cannot access central locations, such as rural communities, often missing out due to lack of awareness of the engagement or an inability to travel to the required location. Ultimately, these associated costs impact on scale and reach as there are limited budgets within departments to run deliberative processes, and there are limited amounts of people within the community that can physically access these interactions.

Governmental usage of online engagements tools such as polling and surveying aims to provide efficiencies and accessibility to community engagements. These techniques can be effective in capturing data. Nonetheless, once collected, government departments and research teams are subsequently burdened with large amounts of qualitative data, sometimes to the point where it is overwhelming or unmanageable. This is also true for deliberative processes that capture transcriptions from face-to-face interactions. This in turn creates flow-on barriers such as potentially prohibitive costs required to resource analysis, excessive time to respond to input from respondents and lack of nuance in the insights that can surface important issues in the data. Additionally, the benefit of NB arises when participants are challenged to balance conflicting values and interests with diverse groups, discussing trade-offs and negotiating mutually agreeable outcomes. These interactions are dynamic and often occur in real-time [86], which is hard to achieve with point-in-time approaches such as surveys and polls.

Any potential solutions that seek to reduce the described cost barriers of deliberative processes must address two core issues. Firstly, they must provide alternatives to physical face-to-face interactions that offer better scale and reach so greater participation can be achieved throughout communities. Secondly, effective and efficient ways to capture, manage and analyse data are required in order to reduce administrative burden and decrease the time it takes to respond to participants and stakeholders involved in the deliberative process.

Regarding qualitative data, it is often important in a deliberation process to extract and understand topics that are being discussed. A primary challenge to achieving this can arise if the volume of data acquired is large. With a relatively small number of submissions, summarising topics and sentiment can be achieved relatively smoothly. When there are hundreds, thousands or even millions of submissions and responses to not only read but to analyse, it becomes unfeasible even with the best qualitative techniques. And that is if the data has been collected at a point-in-time. If there were a dynamic and continual flow of submissions and responses to manage and analyse, the barrier can increase by some magnitude.

### Some ADA technologies able to cope with NB

Developments in NLP, data mining, and machine and statistical learning offer powerful opportunities to help reduce barriers relating to data collection and analysis. Specifically, using computational methods to derive topics provides a potential pathway to help elevate the challenge of large volumes of qualitative data. Some methods include latent Dirichlet allocation [8], probabilistic latent semantic analysis [34], non-negative matrix factorisation [26, 43], recurrent neural networks [5], and transformers [37]. These methods coupled with sentiment analysis, a field of NLP that provides insights to emotional tone behind words, can help provide context behind people’s responses toward a topic [16]. Semantic clustering, another NLP-based technique, has demonstrated that individuals can be organised into factions that expose viewpoints and topics through voting mechanisms, surfacing areas of opposition and consensus [41]. Such an approach could prove very useful as part of the NB process, as understating points of opposition and consensus can help navigate discussions towards trade-offs and outcomes.

The technologies described here are not comprehensive in scope, and most of the methods have specific applications. The next step would involve investigating how a combination of these methods could be integrated with online technologies such as video conferencing and social media to provide face-to-face alternatives. The aim of such investigation would be to research ways to capture and analyse data from these online technologies through a real-time application, with a focus on ameliorating the outlined barriers of cost, time and reach associated with deliberative processes.

### Applying NLP to understand public’s opinion on ADA technologies

An example of narratives around ADA technologies, particularly around AI, is provided by Cave et al. [13]. This collaborative project between the Royal Society and the Leverhulme Centre for the Future of Intelligence is the product of a consultation from communicators, policymakers, and the public regarding their beliefs about AI. Some of the key findings of this project indicate that those not directly engaged with science and technology are susceptible to be influenced by narratives around AI, hence affecting their perception and degree of confidence in those technologies. This, in turn, influences how people apply and use AI-related technologies. The project also found that “prevalent narratives, including misleading ones, can influence policymakers: they either respond to these narratives because these are the ones that resonate with the public, or they are themselves influenced by them” [13], p. 15.

In the Australian context, surveys on people’s understanding and expectations in relation to AI (e.g., [48]) and data governance [7] indicate that there is low trust in AI and that there is low confidence in the government’s handling of people’s data. The study of Lockey et al. [48] also found that one of the factors affecting people’s trust in AI is the degree of familiarity and understanding of AI (these findings have been corroborated and extended in other four developed countries [30]. Such finding resonates with those by Cave et al. [13] regarding people’s narratives around AI. Indeed, one of the mandates of the Australian’s ‘AI action plan’ is to develop and use “AI technologies responsibly to address national problems, build competitive businesses, and increase our collective wellbeing” (Australian Government Department of Industry, Science, Energy and Resources [2], p. 11). Some of the ways to address such mandate at a society level are achieved by promoting awareness and understanding of AI and encouraging safe adoption of human-centred and trustworthy AI [29]. The results of these reports thus indicate that governments should promote dialogue and debate with the public about AI and more broadly about ADA technologies, as these technologies are already in place and developing rapidly.

Indeed, ethical governance is a precursor to public trust in AI. In this regard Winfield and Jirotka [90] propose that such trust will ensue if there are open access ethical codes of conduct, ethics and responsible research and innovation training for everyone, responsible innovation practices, and transparent reports. These recommendations are in line with documents and reports from the European AI Alliance. A key tool developed by the AI HLEG (High-level Expert Group on Artificial Intelligence) to assess trustworthy AI is the ALTAI (The Assessment List on Trustworthy Artificial Intelligence).^13^ Although ALTAI targeted business and organisations, this tool could be used by governments to self-assess the trustworthiness of their AI systems under development. The main challenges relating to ethical AI point to how algorithms process data to produce evidence and motivate actions [58] and how ethical principles translate into practice [59]. Answering those challenges will trickle down to people’s perceptions of ADA.

Hence, our objective is to outline an approach to mine people’s perceptions of ADA technologies as these account for the inseparable triad; algorithms, data, and AI. This is achieved by adopting an NB approach, boosting it with NLP algorithms, supplementing it with semantic differential rating scales, and adding expert knowledge elicitation (EKE) techniques. All these elements are to be deployed via an online platform (OP). The OP serves to collate ideas and opinions. While there are NLP techniques to parse language data, the OP could be supplemented with interfaces that allow obtaining more nuanced data from the community. For example, when a person is asked to type his opinion to the question ‘what is your understanding of the concept of ‘algorithm’?”, there could be a slider rating scale to answer a related question such as ‘how confident are you with your response?’.^14^ This type of semantic differential scale allows to weigh up the participant’s response to the first question. The rating method can also be used to answer to questions that read as open-ended. For example, instead of asking ‘would you trust your government in using ADA technologies to make policies?’. The same question could be rendered as ‘how much trust would you have on your government in using ADA technologies to make policies?’ and the person answers on a slider rating scale anchored by ‘very low level of trust’ (left end of the scale) and ‘very high level of trust’ (right end of the scale). The idea behind the ratings is to supplement open-ended questions amenable to NLP analysis with questions that output numbers. A combination of both categorical (i.e., written text given by open-ended questions) and numeric data (via ratings) would enable to (i) have more information around the topic of interest and (ii) build explanatory and predictive statistical models suitable for informed decision-making. Other demographic data (e.g., age, gender, socioeconomical status) could be captured too to be incorporated into statistical models. Additionally, questions that relate to selected psychological aspects of the respondents (e.g., measures of their soft and critical thinking skills and data literacy) and that are relevant to the topic of interest could be implemented in such survey, if deemed necessary.^15^

### The relevance of expert knowledge elicitation (EKE)

While NLP enables parsing large amounts of text and semantic differential rating scales enable to capture quantitative data, EKE is a technique to extract knowledge from human experts in relation to a topic of interest [4, 65, 78]. Some of the classic EKE techniques are interviews, protocol analysis, concept sorting, among others [78]. Other approaches based on statistics, probability, and decision-making theories, elicit quantitative knowledge by obtaining information about an uncertain quantity from a person’s subjective experience and converting it into a probability distribution [4, 33, 65]. Each EKE approach can be used for categorical (e.g., interviews) or numeric topics (e.g., via subjective probability distributions) but the key feature of EKE is that it allows eliciting judgments from people in a highly systematic and scientific fashion.

Formal protocols for EKE were developed for eliciting quantitative information. Expert elicited data can however be prone to cognitive and contextual biases. Since most often expert judgements are used in the same way empirical data is used, the same level of rigor when eliciting expert judgements is needed. Methods for doing so are called structured protocols (e.g., [33]) and aim to ensure that judgements are reliable and open to the same level of scrutiny as any other forms of data. Most structured protocols recommend using more experts and eliciting individual reasons and rationales behind the numerical estimates.

It is typically considered best practice to elicit judgements from diverse groups (e.g., [11, 69]) such that experts can provide different perspectives, cross-examine each other’s reasoning, and share information. Different protocols provide feedback in different ways (anonymous, through a facilitator who coordinates the flow of information, or through face-to-face discussion) and allow interaction and discussion to different degrees.

These differences are closely related to the preferred aggregation of group judgements approach (given that usually one unifying set of estimates is needed in the following analysis or in the decision process). When behavioral aggregation is preferred (where the group strives for consensus) the discussion stage needs to be extensive and continue until consensus is reached (e.g., O’Hagan et al. [66]). When mathematical aggregation is chosen, different protocols propose different strategies. The classical model for expert judgement [17] allows very little interaction and no feedback on the provided rationales. These are only used by the analyst in the aggregations stage. The IDEA protocol (e.g., [32]) however uses the initial round of estimates and rationales as a starting point for a discussion stage. IDEA stands for Investigate, Discuss, and Estimate, and Aggregate. For each question to be answered, experts engage in an individual and independent research on background information and investigate related sources of information; provide anonymous numerical estimates, together with their justifications; receive feedback that reveals how their individual estimates differ from others’ (a plot of all group members' estimates, together with their justifications); discuss differences in opinion and ‘consider the opposite’; and finally, provide a second anonymous estimate of the probability, incorporating insights gained through feedback and discussion.

A recent application of the IDEA protocol which takes most advantages of the qualitative data set elicited together with the quantitative estimates is developed by the repliCATS team (https://replicats.research.unimelb.edu.au/) and it is part of the SCORE program (Systematizing Confidence in Open Research and Evidence) funded by DARPA (Defence Advanced Research Projects Agency) in the US. It is one of the largest replication projects in history which aims to develop tools to assign “confidence scores” to research results from the social and behavioural sciences. The qualitative data set is analysed and used to reward the breadth and diversity of reasons provided to support the individuals’ estimates. These rewards are then used as weights in a differential weighing aggregation scheme. To the best of our knowledge, this is the first proposal of a mathematical aggregation (linear opinion pool) that uses the elicited qualitative data.

**Fig. 3:**
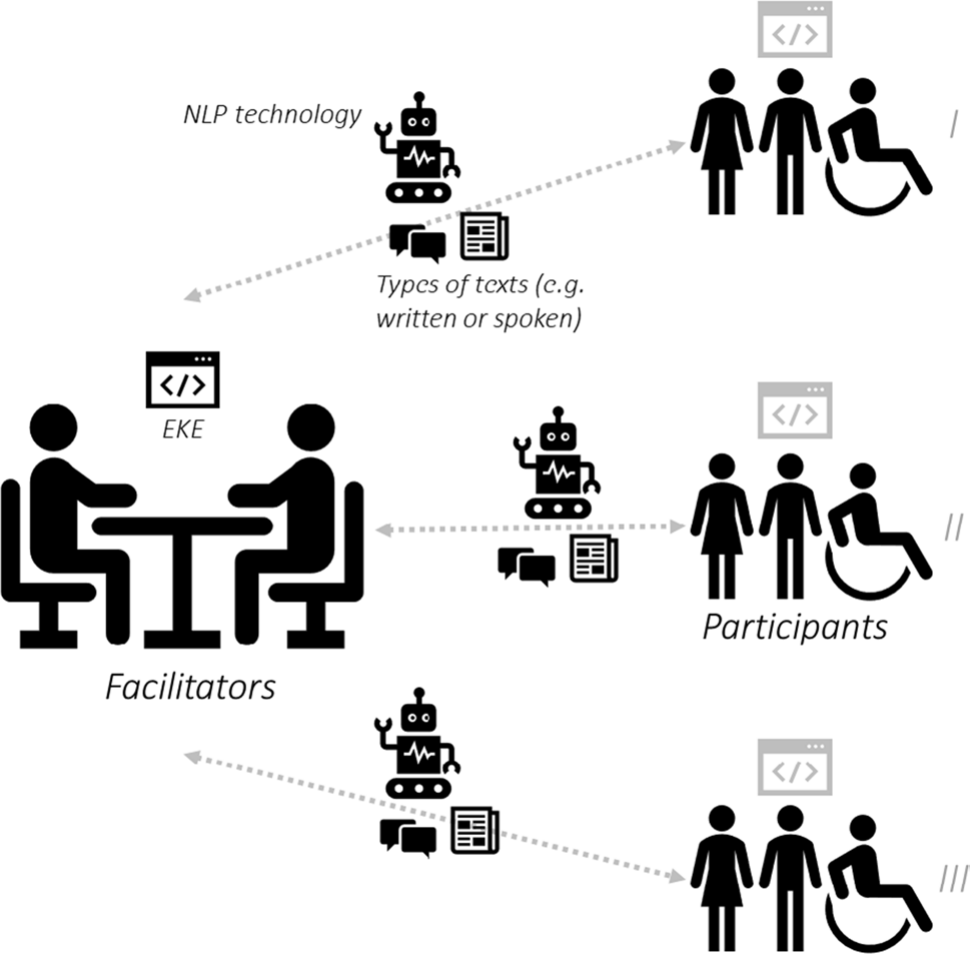
Illustration of an AI-powered NB method. The numerals *I*, *II*, and *III* represent the order of steps in NB (see Fig. 2). The grey icon above ‘participants’ indicates that EKE could also be performed at that stage in the process (e.g., EKE could be used with the participants to produce more cohesive texts). Facilitators use EKE to further parse the NLP outputs and thus arrive at highly informed decisions and results

Based on evidence suggesting that combining expert knowledge with machine learning’s outputs leads to better predictions (e.g., [19, 79]), our proposal thus incorporates EKE within the AI-powered NB approach. Figure 3 illustrates our proposal for AI-powered NB. Note that although the model was conceived having in mind the goal of mining public’s perceptions of ADA technologies, it is applicable to other topics. Also, the proposed method incorporates principles of human-in-the-loop at the ‘facilitators’ level via the EKE step in that NLP’s outputs are further evaluated by humans (i.e., the facilitators). A degree of society-in-the-loop is conceivable if the ADA technologies used in the NB process are made transparent and explainable to the parties involved; e.g., issues related to security, privacy, fairness, etc. [74].

## Discussion

In this article, we introduced the term “narrative building” as a pragmatic way to facilitate public engagement with digital governance initiatives. As an instrumental tool for maximising the chances of DG initiatives to be successful, we claimed that this narrative building can be enhanced by incorporating expert knowledge elicitation and natural language processing capabilities along with semantic differential rating scales. To make this case, we illustrate how this approach could be used in the case of capturing the public’s opinion on ADA technologies. The key messages are that (i) as narratives are natural forms of human cognition, NB is thus a seamless tool to appeal public participation and engagement, (ii) that powering NB with ADA technologies such as NLP, allow NB to have reach and scale, and (iii) that the implications for governance translate in boosting public trust as NB is about capturing public’s opinion. This last point is in line with most governments’ goals of promoting awareness and understanding of AI and encouraging safe adoption of human-centred and trustworthy AI. Below we emphasise some aspects related to our proposal.

A key aspect we would like to stress is that our proposal has implications for governance. By harnessing EKE with NLP, the proposed approach ensures data processing is expedited as it is co-handled by AI-related technologies and not solely by humans. The use of NLP technologies has indeed several advantages. For example, NLP could help attorneys and lawyers to understand if a new regulation proposal conflicts with previous o related regulations, which in turn will reduce the process of discussing and approving regulations at the congress level. Likewise, public servants will have less excuses for delays in legal decisions toward criminal offenders’ trials. Moreover, conflicts of civil rights (e.g., divorces with infant custody claims) could be solved more rapidly. Finally, in terms of planning for the future, EKE + NLP technologies could help decision-makers in the public sector understand key factors in societies’ problems (e.g., urban waste management, public transportation, import taxes, public health attention).

Within the NB approach, narratives are built by (i) setting a common ground via (ii) a bottom-up approach. The first part relates to concept generation and their clustering in groups of semantic similarity, while the second part indicates that step (ii) requires the participation of the community. A feature of NB is that there should be agreed rules to regulate interactions between facts and values intrinsic to the complexity of the issue at hand. Once clusters of concepts or topics around the issue of interest are identified (i.e., a master narrative and its sub-narratives; here called Nm and Nsn), a second tier of review is carried out via focus groups (i.e., community engagement groups). A final step is to validate the Nm and its Nsn with members of the community (see Fig. 2). Although NB was represented via three orderly steps, iterations within and between steps are not excluded. Indeed, the entire NB process can the conceived as a cyclical construction of narratives such that the steps I > II > III can be iterated (see Fig. 3).

The current proposal is conceived for deliberation situations that can afford ample timeframes (e.g., weeks to months), and it is not clear how the method would unfold in the case of deliberation situations that need very fast turn arounds where time is of the essence (e.g., hours to days). It is important to note too that the government reports cited above in relation to people’s understanding of AI and data have relied on traditional categorical *n*-point Likert scales with no open-ended questions and data relating to participants’ psychological factors. As shown above, our proposal improves such traditional approach in several ways. For example, by incorporating the power of NLP techniques, the proposal enables scale and reach. Also, NB by default is a tool that promotes public engagement. We believe that if the model were applied to the case of public’s perception of ADA technologies, the outcome would contribute to ethical governance around those types of technologies. An empirical implementation of this proposal and its implications to ethical governance of ADA technologies have yet to be explored.

## Footnotes

1 Throughout this manuscript any reference to ‘algorithms’ is to be understood as ‘machine learning algorithms’. That is, algorithms represent the machine learning techniques that are used to get insights from data and AI is understood as a trained model ready to use (see Sect. 4 for details).

2 https://www.opengovpartnership.org/policy-area/digital-governance/#overview.

3 Australia Action Plan 2018–2020 (https://www.opengovpartnership.org/documents/australia-action-plan-2018-2020/).

4 Which is the OPG recommendation to France via the France Action Plan 2018–2020 (https://www.opengovpartnership.org/wp-content/uploads/2018/08/France-Action-Plan-2018-2020-English.pdf).

5 Which is the OPG recommendation to Canada via the Canada Action Plan 2018–2020 (https://www.opengovpartnership.org/wp-content/uploads/2019/01/Canada_Action-Plan_2018-2020_EN.pdf).

6 Predictive and Decision-Making Algorithms in Public Policy QSD on 12 February 2020; https://lordslibrary.parliament.uk/research-briefings/lln-2020-0045/,

7 https://www.partnershiponai.org/report-on-machine-learning-in-risk-assessment-tools-in-the-u-s-criminal-justice-system/.

8 https://www.opengovpartnership.org/documents/germany-action-plan-2019-2021/.

9 https://www.opengovpartnership.org/documents/colombia-action-plan-2020-2022/.

10 Disclosure of code is a controversial topic though. For example, it can be questioned whether disclosure of source code would provide sufficient information to satisfy the principle of accountability during decision making. That is, to understand how a decision was made the citizen does not need to see the source code. Indeed, even if the citizen sees the source code it does not readily imply that he will understand how the decision was made. In some cases, source codes are proprietary, and this prevents open and public access. A potential solution that bypasses source code disclosure would be to provide general information about the algorithm, its goals, and some auditing that ensures its legal and technical compliance.

11 An example of how a narrative structure can be used to communicate expository information is the book ‘An adventure in statistics’ (SAGE, 2016) by Andy Field. This book was shortlisted for the Association of Learned & Professional Society Publishers Award for Innovation in Publishing 2016 and it blends key concepts in statistics with visual story-telling. This book then emphasises critical thinking and problem solving over rote memorization.

12 Another example of the relevance of narratives as a tool to give meaning to any topic is that of ‘narrative visualisations’. In this case, narrative structures are used to better grasp data visualisations to enhance comprehensibility by evoking emotional reactions in the user [57, 77].

13 It is worth noting that the European AI Alliance is not the same group as the AI HLEG. The initial scope of the Alliance was to provide feedback to the AI HLEG, which, in turn, was appointed by the European Commission to assist with AI policy development. Currently, the Alliance works as a multistakeholder platform and the AI HLEG has concluded its activities.

14 A slider rating scale can be customised to take values with, say, two decimal places between, say, the values 0 and 10 where only the values 0 and 10 are labelled via two polar adjectives. Statistically speaking, this type of rating scale outputs more fine-grained results than traditional *n*-point Likert scales. Note also that numerical scales should be properly defined to avoid cardinal/ordinal confusions.

15 The responses given by a person to the survey are necessarily tied up to his/her socio-cognitive skills; hence some measure of the participants’ psychological traits and states is necessary to produce richer interpretation of the results. Although there is evidence that digital footprint (e.g., social media activity) can be used as a proxy to approximate personality factors [22, 40, 53, 80, 83]. There is also a special issue ‘behavioural personality science in the age of big data’ in European Journal of Personality), using existing psychometric surveys is a more direct and reliable approach.
